# An ultrasonic sensing system for colonic content discrimination in ostomy patients: Development and experimental validation

**DOI:** 10.1371/journal.pone.0361870

**Published:** 2026-11-11

**Authors:** Yongshi Tang, Yi Pan, Tianjun Huang, Yan Li, Shanjiao Zhong, Hui An, Guangfei Deng, Xiongbo Guo

**Affiliations:** 1 Department of Gastrointestinal Surgery, The Second Affiliated Hospital of Guangzhou Medical University, Guangzhou, Guangdong Province, China; 2 Department of Pathology, Guangzhou Panyu District Maternal and Child Health Hospital (He Xian Memorial Hospital), Guangzhou, Guangdong Province, China; 3 Operating Room, The Second Affiliated Hospital of Guangzhou Medical University, Guangzhou, Guangdong Province, China; 4 Department of Pathology, School of Basic Medical Sciences, Guangzhou Medical University, Guangzhou, Guangdong Province, China; Government Gousia Hospital, DHS, Srinagar, INDIA

## Abstract

**Background:**

Enterostomy leads to excretion incontinence, complications, and impaired quality of life, while existing interventions lack colonic content sensing capabilities.

**Objective:**

The aim of this validation-based study was to develop and evaluate the technical performance of a smart ultrasonic sensing system for discriminating colonic content states to improve ostomy management.

**Method:**

A modified 2 MHz reflective ultrasonic sensing system was developed, integrating an ultrasonic probe, transceiver circuit, signal amplification module, single-chip microcomputer, RGBW LEDs, and a buzzer alarm. Functional testing was performed under three immersion conditions (non-immersed, semi-immersed, and fully immersed) in normal saline. Voltage thresholds were established in vitro using excised colonic segments from 20 *Oryctolagus cuniculus* (*O. cuniculus*), with voltage signals measured under four colonic content states (gaseous, solid, fluid, and empty). In vivo validation was then conducted in 20 *O. cuniculus* and 12 *Canis lupus familiaris* (*C. lupus familiaris*). Data were analyzed using one-way ANOVA with LSD post-hoc tests (SPSS 27.0, p < 0.05). System performance was evaluated by the concordance between LED and buzzer outputs and reference colonic content states.

**Results:**

Voltage output increased progressively with immersion depth in normal saline: 343.85 ± 2.11 mV (non-immersed), 637.60 ± 8.01 mV (semi-immersed), and 943.35 ± 3.86 mV (full- immersed) (*p* < 0.001). In vitro, distinct voltages were recorded across states: gaseous (257.12 ± 10.51 mV), solid (398.12 ± 32.33 mV), fluid (507.24 ± 25.01 mV), and empty (658.56 ± 14.85 mV) (all p < 0.001). In vivo, the system achieved 98.0% overall accuracy in *O. cuniculus* (392/400) and 97.0% in *C. lupus familiaris* (419/432), with the solid state showing the lowest accuracy.

**Conclusion:**

This study developed an integrated ultrasonic sensing system for artificial anal plugs, which achieved high classification accuracy and can provide reliable real-time feedback on colonic content, demonstrating strong potential for artificial anal plugs.

## Introduction

Colorectal cancer represents the primary cause of enterostomy [1,2]. The formation of an enterostomy not only exposes patients to a variety of physical complications, such as stoma retraction, necrosis, prolapse, stoma-related leakage, and peristomal skin complications [3–5], but also may lead to deterioration in mental health resulting from physical and social changes [6]. Current studies have demonstrated that concerns regarding the control of excreta constitute the primary worry among enterostomy patients [7]. The uncontrollability of excreta, odor, and associated noise, coupled with the embarrassment arising therefrom, often prompts patients to restrict their activities to avoid awkward situations in public settings [8,9]. Thus, there is an urgent clinical need for innovative solutions to address enterostomy patients’ demand for restored defecation control, thereby alleviating ostomy-related complications and improving their quality of life.

For a long time, scholars have explored solutions to help enterostomy patients solve these problems such as pelvic floor muscle training [10], biofeedback training [11] and nerve stimulation [12], but efficacy is limited. A colostomy plug, invented in 1986, marked a breakthrough [13]. It expands upon contact with water to block fecal passage, yet it imposes long-term financial burdens on patients and carries inherent complication risks. Subsequent plugs improved fecal incontinence management but still have flaws: no colonic content sensation, strong sense of discomfort, high risk of dislodgement and leakage, and poor adjustability [14–17]. Colonic content perception is essential for enabling patients to recognize intestinal status, regulate defecation voluntarily, and avoid unexpected leakage or discomfort, making its absence a major barrier to improved quality of life. This highlights the critical need for an intelligent sensory system that prioritizes the detection of colonic content, offering a more effective and comfortable solution.

Ultrasound is a mechanical pressure wave propagating through elastic media [18], which is ideal for sensing due to its focused propagation, high energy, strong directionality, and good penetration [19]. The propagation of ultrasound is influenced by acoustic impedance, which varies with the density of the medium [20]. Therefore, when ultrasound travels through media with different densities, distinct signals are generated. These signals can be converted into voltage signals by sensors, enabling the differentiation of these materials. Based on this principle, ultrasonic sensing technology can qualitatively identify colorectal contents by capturing signal variations induced by density differences among intestinal materials. This capability offers technical support to address the sensory deficit inherent in conventional artificial anal plugs.

Building on this acoustic foundation, we designed a novel sensing system for colonic content discrimination using modified reflective ultrasonic technology. This system is a smart ultrasonic monitoring device that integrates a 2 MHz transceiver with real-time signal processing capabilities. By analyzing acoustic impedance variations, the system identifies four distinct colonic content states (gaseous, solid, fluid, and empty) and delivers immediate sensory feedback through visual and auditory alerts. Addressing the sensory deficit of conventional ostomy devices, the system offers a non-invasive, real-time monitoring solution that potentially enables autonomous defecation control for ostomy patients.

The aim of this validation-based study was to develop and evaluate the technical performance of the proposed sensing system for discriminating colonic content states. We hypothesized that (i) distinct and separable voltage signatures would be observed in vitro across the four colonic content states, enabling the definition of clear decision thresholds, and (ii) implementation of these thresholds would allow accurate and reliable classification of colonic content states during in vivo testing in animal models, as reflected by the LED and alarm outputs.

## Materials and methods

### 1. Animals

All experiments were conducted in compliance with the *Guiding Opinions on the Humane Treatment of Experimental Animals* (2006) issued by the Ministry of Science and Technology of the People’s Republic of China and were approved by the Ethics Committee of Guangzhou Medical University (Guangzhou, China). All procedures were conducted in accordance with institutional animal welfare guidelines, and humane endpoints were predefined.

Forty adult *Oryctolagus cuniculus* (*O. cuniculus,* New Zealand white rabbits, body weight: 2.5 ± 0.2 kg, both sexes) and twelve adult *Canis lupus familiaris* (*C. lupus familiaris,* Beagle dogs, body weight: 12.5 ± 2.5 kg, both sexes) were provided by the Animal Experimental Center of Guangzhou Medical University (Guangzhou, China; License No. SCXK (Yue) 2006−0015) and used for the experiments.

All animals were housed in a controlled environment at 22 ± 2°C with a 12-hour light and dark cycle. Animals were acclimated to the laboratory environment for at least 7 days before experiments. During the acclimation period, animals were fed standard laboratory chow and had free access to water. Feeding regimens during experimental periods were specifically designed to induce different colonic content states, as described in the experimental protocols below.

All surgery was performed under anesthesia, and all efforts were made to minimize suffering. Anesthetic depth was monitored throughout the procedures. At the end of terminal procedures, animals were euthanized under deep anesthesia by intravenous administration of an overdose of a barbiturate anesthetic, and death was confirmed by cessation of respiration and heartbeat.

### 2. Design and components of the smart sensing system

The ultrasonic signal detection system, a modified version of the TCT40–18FS-1.2B detector, features a transmitting frequency and an ultrasonic probe frequency both set to 2 MHz, and was equipped with a two-stage amplification circuit with sequential magnification factors of 48× and 35 × .

Fig 1 illustrates the physical appearance and specific mechanical dimensions of the modified, fully sealed integrated ultrasonic probe. Two distinct pin configurations with 10-mm (Fig 1A, a and 1B, a) and 5-mm (Fig 1A, b and 1B, b) pitches are presented to accommodate different spatial assembly and printed circuit board layout requirements. In the final device prototype used for animal experiments, the 10-mm pin pitch probe was ultimately selected.

**Fig 1:**
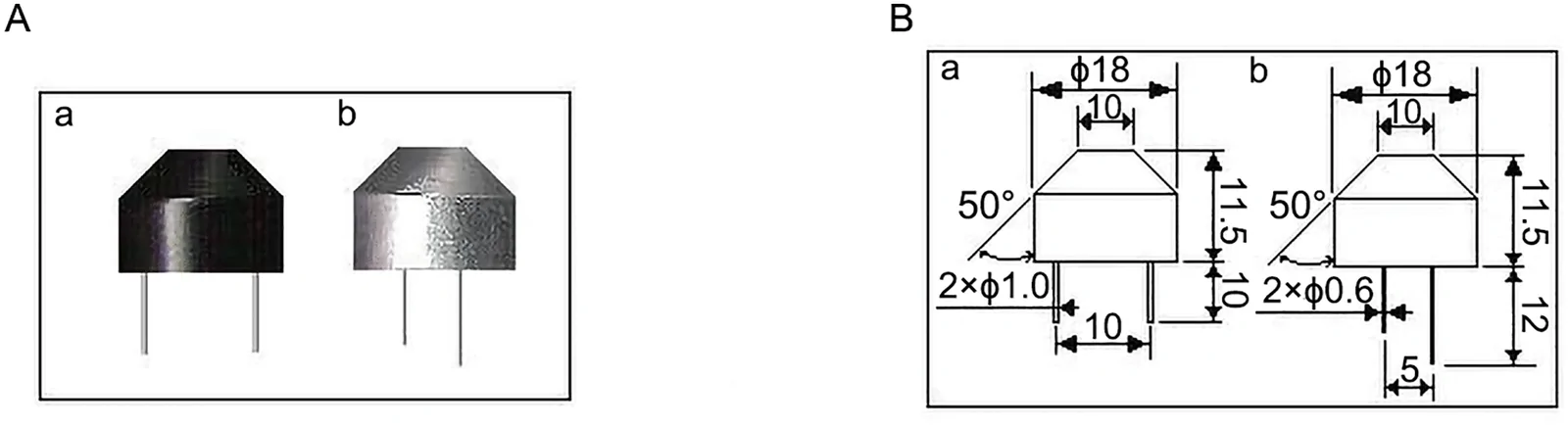
Physical appearance and mechanical specifications of the integrated ultrasonic probe. (A) 3D renderings of the fully sealed modified transducers, showing two variants (a, b). (B) Mechanical blueprints detailing the uniform housing dimensions with two distinct pin configurations: (a) a 10-mm pitch layout and (b) a 5-mm pitch layout.

The core experimental device consisted of six key components (Fig 2): (A) a power supply (SendK 5V direct current power supply, XingBoNeng Science and Technology Co., LTD, Shenzhen, China); (B) an integrated ultrasonic probe (Modified TCT40–18FS-1.2B transducer); (C) integrated circuits (including ultrasonic transmitting circuit, receiving circuit, rectifier circuit, first and second-order filter circuits); (D) a single-chip microcomputer; (E) an alarm system (comprising a buzzer with a vibrator) and (F) RGBW (red, green, blue, white) light-emitting diodes (LEDs, provided by South China University of Technology).

**Fig 2:**
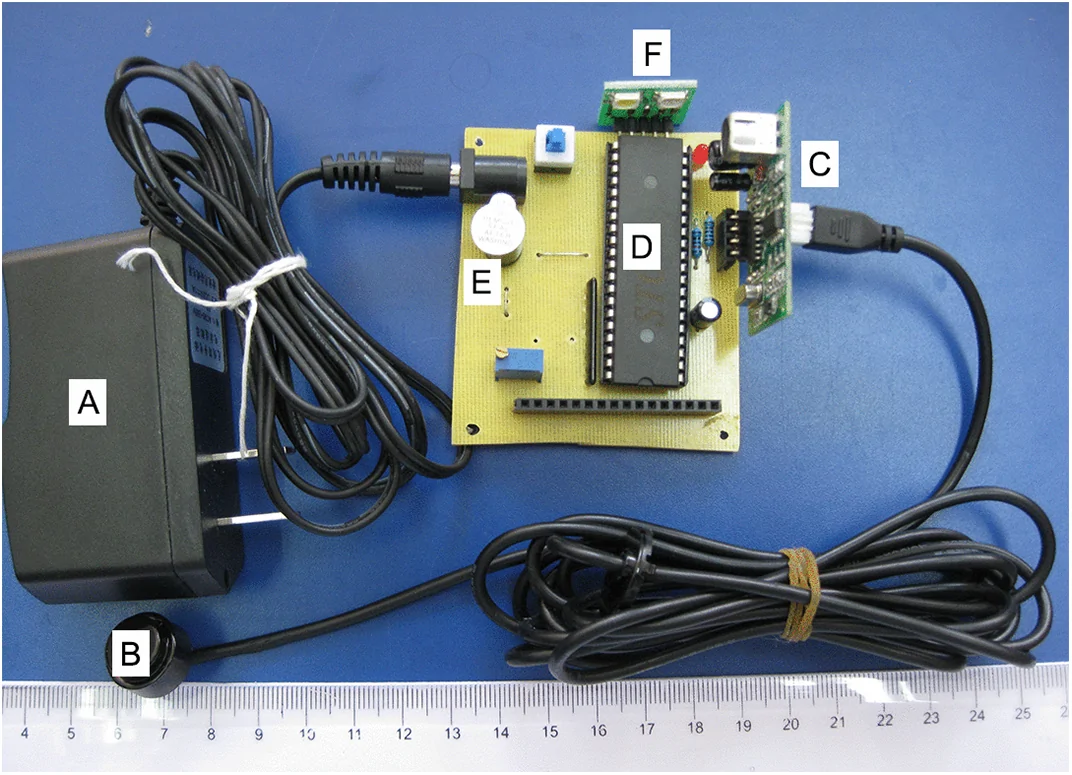
Physical diagram of the smart sensing system for artificial anal plug. (A) Power Supply (SendK 5V direct current). (B) Integrated Ultrasonic Probe (modified TCT40–18FS-1.2B, 2 MHz frequency). (C) Integrated Circuit (including transmitting, receiving, rectifier, and filter circuits). (D) Single-Chip Microcomputer. (E) Alarm System (buzzer and vibrator). (F) LEDs.

### 3. The smart sensing system development and validation

The development and validation of the smart sensing system followed a multi-stage experimental design. The process began with functional testing, followed by the determination of voltage thresholds using an in-vitro-excised *O. cuniculus* colon model. These thresholds were implemented into the system’s software algorithm. Finally, the performance of the complete system was validated sequentially in *O. cuniculus* and C. lupus familiaris in-vivo models.

#### 3.1 Functional testing of the smart sensing system.

To verify the basic functionality and sensitivity of the ultrasonic sensor prior to biological experiments, the integrated ultrasonic probe, equipped with a waterproof protective cover, was tested using 20 cups of normal saline maintained at 38.5°C. In each trial, five consecutive voltage measurements were recorded, and the mean of the five measurements was used as one data point. The test was performed under three states (n = 20 per state, total = 60 data points). To measure voltage, a DT9205 digital multimeter (Focus Instruments Co., Ltd., Shenzhen, China) was connected to the circuit, with the multimeter range set to 2V. The three test conditions were:

(1) **Non-immersed group**: Probe placed in air without contact with normal saline.
(2) **Semi-immersed group**: Half of the probe’s surface immersed in normal saline.
(3) **Fully immersed group**: Probe completely immersed in normal saline.

#### 3.2 In-vitro voltage measurement for threshold determination.

Twenty *O. cuniculus* were anesthetized via ear vein injection of 20% urethane (5 mL/kg), followed by routine disinfection and draping. The abdominal cavity was exposed through a midline incision, and a 10 cm colonic segment located 20 cm proximal to the anal verge was subsequently identified and excised. The excised colonic segment was transversely transected, and its contents were gently squeezed out. The four states of colonic contents were prepared as follows:

(1) **Empty state**: All original contents in the isolated intestinal segment were expelled one by one. Ultrasonic probe of the reflective sensor was inserted into the transected end of the colon, and the end was then clamped.
(2) **Gas state**: A three-way tube was connected to a 10 mL syringe and a needleless scalp acupuncture tube. The scalp tube was inserted into the colon, and 10 mL of air was injected.
(3) **Solid state**: Air in the colon was evacuated, and fecal pellets were pushed from the proximal to the dissected colon until they reached the ultrasonic probe.
(4) **Fluid state**: Fecal pellets in the proximal colon were gently compressed, 5 mL of normal saline was instilled into the colon, and the mixture was homogenized to form a fluid.

Fig 3 shows the different states of colonic content in the excised segments.

**Fig 3:**
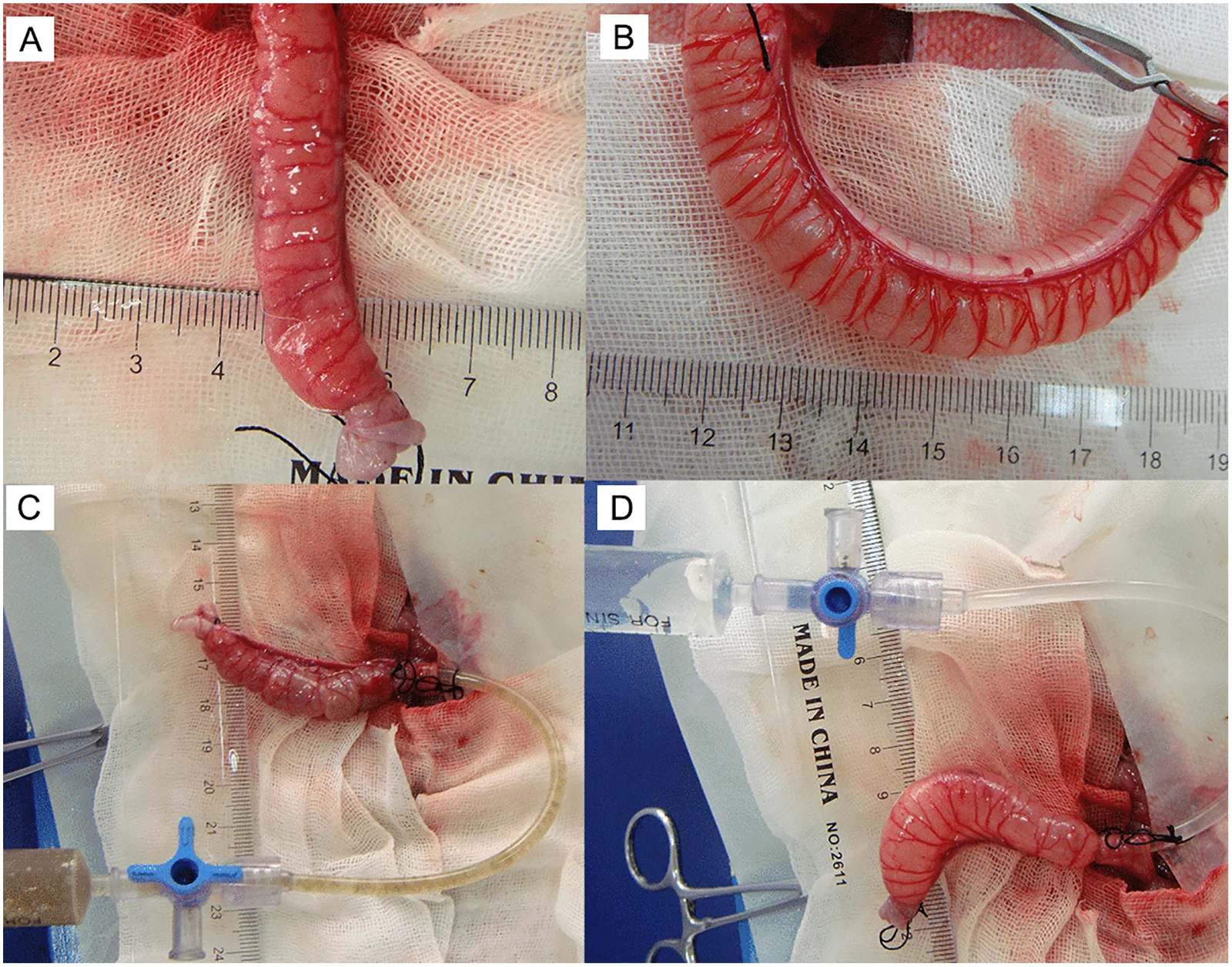
Different states of colonic content in excised segments. (A) Empty state: All contents removed, ultrasonic probe inserted and clamped. (B) Gas state:10 mL air injected via scalp tube. (C) Solid state: Fecal pellets pushed to the ultrasonic probe. (D) Fluid state: Fecal pellets mixed with 5 mL normal saline.

After the content state stabilized, the SendK 5V direct current power supply was turned on, and voltage was measured using a DT9205 digital multimeter connected to the circuit. For each excised segment in each content state, the state preparation and measurement were repeated in five trials; in each trial, five consecutive voltage readings were recorded and averaged as one data point, yielding 100 data points per state across 20 segments (n = 100 per state, total = 400 data points).

To ensure reliable state discrimination and minimize misclassification risk, voltage threshold cutoffs were designed to be positioned at intermediate points between adjacent states. The specific threshold values were determined based on the voltage distributions obtained from these experiments, with final threshold and results values presented in the Section 3.3 and Results section.

#### 3.3 Software development and threshold implementation.

All software was programmed in C++ using ICC 7.0 as the development tool. Based on the threshold values determined from in-vitro voltage measurement, the software encoded the voltage cutoffs for automatic state discrimination as follows:


$$\begin{array}{c} \#defineVGASEOUS\;\;((295\times 400+500)/1000)\;//\leq 295\;mV:gaseousstate \end{array}$$

$$\begin{array}{c} \#defineVSOLID\;\;((455\times 400+500)/1000)\;//295-455\;mV:solidstate \end{array}$$

$$\begin{array}{c} \#defineVLIQUID\;\;((598\times 400+500)/1000)\;//455-598\;mV:liquidstate \end{array}$$

$$\begin{array}{c} //>598\;mV:emptystate \end{array}$$

Based on these defined thresholds, the system’s input voltage triggers a unique response from the alarm module and RGBW LEDs to indicate the corresponding colonic state:

**(1) Gaseous State (U ≤ 295 mV):** Green LED on, single ‘beep’ from the buzzer, vibrator activated.
**(2) Solid State (295 mV < U ≤ 455 mV):** Blue LED on, double ‘beep’ from the buzzer, vibrator activated.
**(3) Fluid State (455 mV < U ≤ 598 mV):** Red LED on, ‘urgency’ sound from the buzzer, vibrator activated.
**(4) Empty State (U > 598 mV):** White LED on, buzzer and vibrator inactive.

The overall working principle of the smart sensing system is illustrated in Fig 4.

**Fig 4:**
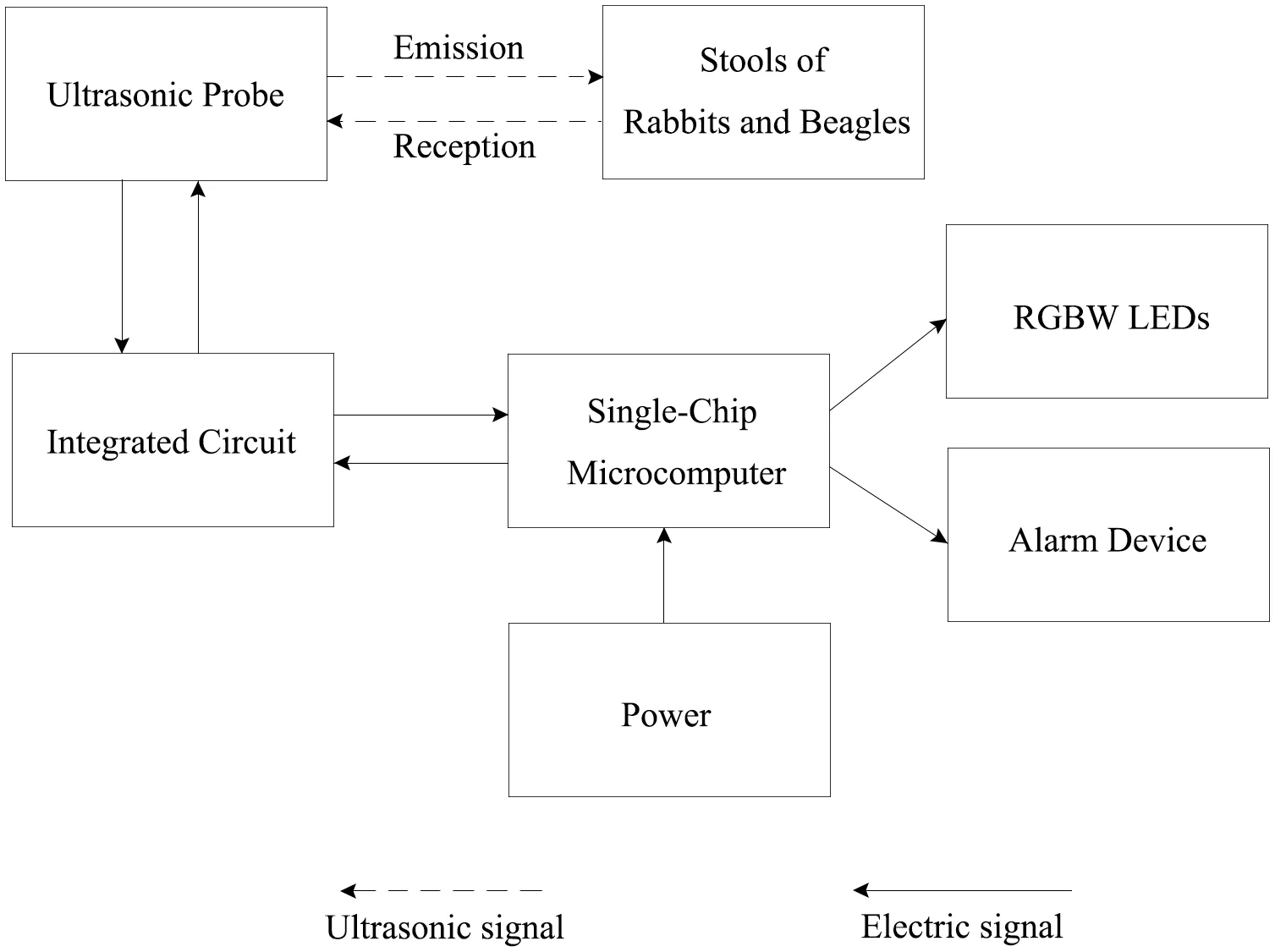
Working principle of the smart sensing system for artificial anal plug.

The Ultrasonic Probe emits and receives signals from stools of *O. cuniculus* and *C. lupus familiar*is. This signal is converted to an electric signal and processed by the Integrated Circuit. The data is then sent to the Single-Chip Microcomputer for analysis. Based on the analysis, the microcomputer activates the output components: the RGBW LEDs for visual feedback and the Alarm Device for an auditory alert. The entire system is powered by a central power source. Dashed lines indicate the path of ultrasonic signals, and solid lines represent the flow of electric signals.

#### 3.4 In-vivo validation of the smart sensing system’s performance.

The system’s performance was validated in two sequential in-vivo stages:

**(1) *O. cuniculus* model**: Twenty *O. cuniculus* were prepared using the same anesthesia and surgical procedures in Section 3.2. In this group, however, the colonic segments were left in situ. The four content states (empty, gas, solid, and fluid) were then established following the same protocols, with the sensor inserted into the colon through a small enterotomy.

A measurement repeatability verification was then performed, consisting of taking five separate voltage readings from the sensor for each of the four content states to evaluate the sensor’s measurement stability and consistency in the in-vivo environment. Subsequently, the complete smart sensing system was activated for each state. The resulting LED illumination status and buzzer alarm response were then recorded five times per content state for every animal.

**(2) *C. lupus familiaris* model**: Twelve *C. lupus familiaris* were anesthetized with Sumianxin II (0.10 mL/kg; active ingredient: xylazine hydrochloride) following subcutaneous administration of atropine sulfate (0.05 mg/kg) 15 minutes prior and maintained with isoflurane (induction concentration 3–5%, maintenance concentration 1–3%). After anesthesia took effect, the abdominal area was shaved, disinfected, and covered with sterile surgical drapes. A midline abdominal incision was made to expose the abdominal cavity, and the distal colon that was 20 cm from the anal verge was identified. Reflective ultrasonic probe was then secured at this site with its detection surface facing the intestinal lumen. The probe was fixed symmetrically to the colon wall to prevent displacement during intestinal peristalsis. Connecting wire of the probe was routed externally and connected to the entire control assembly. This unit and a rechargeable lithium battery were integrated into a lightweight neck collar for real-time observation of responses. Surgical incision was sutured layer by layer after confirming proper placement (Fig 5).

**Fig 5:**
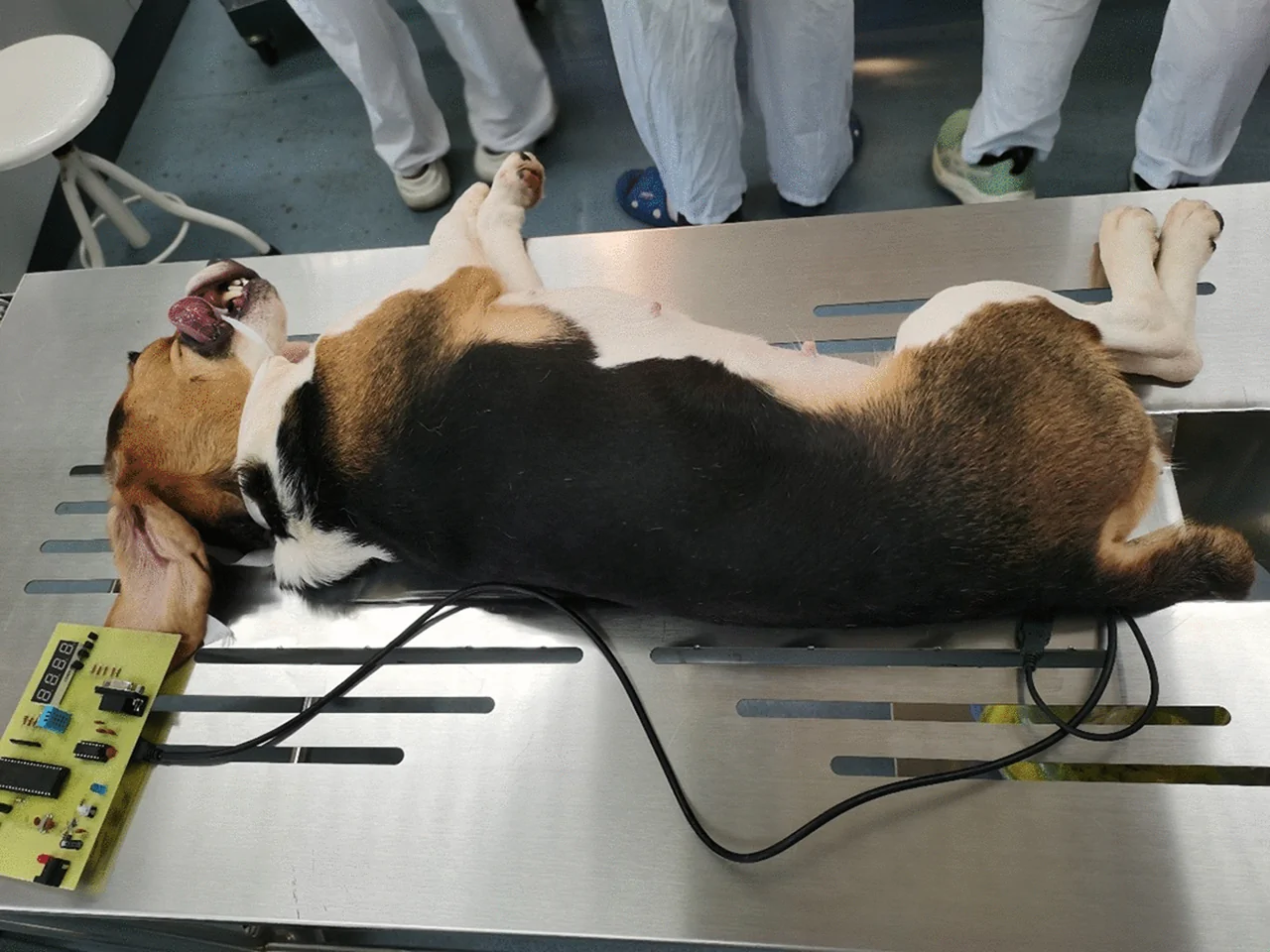
Post-implantation of the smart sensing system in an anesthetized *C. lupus familiaris.*

A reflective ultrasonic probe connects externally to a control assembly (bottom left). The assembly and lithium battery was integrated into a neck collar for real-time monitoring.

After implantation, infection prevention and analgesia were administered, and 5% glucose solution was administered to supplement energy. The four states of colonic contents were prepared as follows:

(1) **Empty state**: Twenty-four hours before testing this state, the *C. lupus familiaris* were fed only water and diluted meat broth. Four hours prior to each detection, 50 mL of normal saline was administered via gastric tube to promote defecation and ensure an empty colon.
(2) **Gas state**: One hour before this test, the *C. lupus familiaris* were fed 50g of gas-producing food consisting of cooked soybeans mixed with standard dog chow, and 10 mL of sterile air was injected into the rectum via a soft catheter to simulate colonic gas retention.
(3) **Solid state**: One day before testing this state, the *C. lupus familiaris* were fed the standardized commercial maintenance dog food at 200–250 g per day to promote the formation of well-formed feces. Abdominal palpation was performed before each test to confirm the presence of solid feces in the colon.
(4) **Fluid state**: Six hours before this test, the *C. lupus familiaris* were fed 200 mL of meat broth, and 50 mL of castor oil was administered via gastric tube to liquefy the intestinal content into a fluid state.

The smart sensing device was activated on postoperative day 7. For each content state, 15–20-min monitoring sessions were conducted three times daily (at 08:00, 12:00, and 18:00) for 3 consecutive days, yielding 9 classifications per state per animal (n = 108 per state, total = 432 classifications).

Ultrasonographic examination was performed synchronously during each monitoring session to acquire real-time images of the colonic content states, which were cross-referenced with the response results of the smart sensing system to evaluate the detection accuracy of the system.

### 4. Statistical analyses

All statistical analyses were performed using the SPSS 27.0 software (IBM Corp., Armonk, NY, USA). A two-tailed *p*-value < 0.05 was defined as statistically significant, and data were presented as mean ± standard deviation (mean ± SD).

For voltage measurements across different colonic content states, one-way analysis of variance (One-way ANOVA) was employed to compare means among the four groups (empty, gas, solid, and fluid). Prior to one-way ANOVA, normality was assessed using the Shapiro-Wilk test and homogeneity of variance was evaluated using Levene’s test. When significant overall differences were detected (*p* < 0.05), post-hoc pairwise comparisons were performed using the least significant difference (LSD) test to identify which specific content states differed significantly from one another. Considering the critical need to detect meaningful differences between adjacent voltage ranges for accurate threshold determination, the LSD test was applied to provide adequate statistical power for these comparisons.

For the classification performance of the smart sensing system in both *O. cuniculus* and *C. lupus familiaris* models, descriptive statistics were employed to report the accuracy of LEDs and alarm responses for each colonic content state. The overall accuracy was calculated as the percentage of correct classifications out of total measurements. Formal statistical comparison of misclassification rates among different states was not performed, as the primary clinical outcome of interest is the overall system accuracy rather than state-specific error patterns.

## Result

### 1. Voltage signals under different immersion

The immersion state experiment (n = 20 per state) revealed a clear and progressive increase in voltage as the sensor’s immersion in normal saline increased, confirming its sensitivity to changes in the surrounding medium. The mean ± SD voltages were 343.85 ± 2.11 mV (95% CI: 342.86–344.84 mV) for the non-immersed state, 637.60 ± 8.01 mV (95% CI: 633.85–641.35 mV) for the semi-immersed state, and peaked at 943.35 ± 3.86 mV (95% CI: 941.54–945.15 mV) for the fully-immersed state (Fig 6, Table 1). One-way ANOVA confirmed a highly significant difference among the three groups (F(2, 57) = 64595.97, *p* < 0.001), and post-hoc LSD tests revealed that all pairwise comparisons were statistically significant (*p* < 0.001).

**Table 1:** Voltage values under different immersion states (n = 20).

State	Voltage Value ($\overset{―}{X}$ ± *S*，mV)	95%*CI*		Minimum Value	Maximum Value	Range
		Lower Bound	Upper Bound			
Non-immersed	343.85 ± 2.11	342.86	344.84	339	348	9
Semi-immersed	637.60 ± 8.01	633.85	641.35	618	657	39
Full-immersed	943.35 ± 3.86	941.54	945.15	937	953	16

F(2, 57) = 64595.97, *p* < 0.001. All post-hoc pairwise comparisons between groups were significant (LSD test, *p* < 0.001).

**Fig 6:**
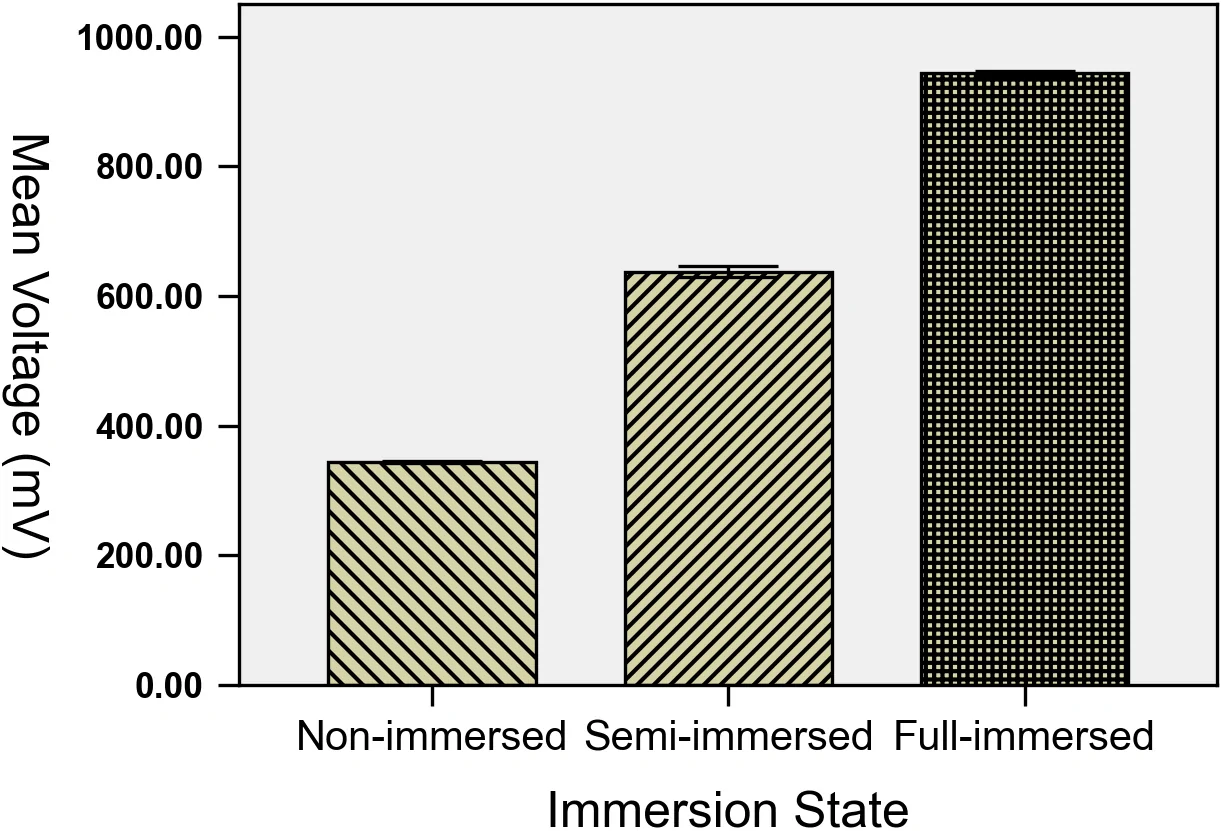
Sensor voltage across different immersion states.

The bar chart displays the mean voltage signals recorded from the sensor under three conditions: non-immersed in air, semi-immersed, and full-immersed in normal saline. Error bars represent the 95% confidence interval (CI). Measurements were performed 20 times for each state (n = 20). Statistical analysis revealed significant differences between all groups (*p* < 0.001).

### 2. In-vitro voltage measurement of colonic content states

The voltage signatures of different colonic contents were measured in an in-vitro model using excised O. cuniculus colons (n = 100 per state). A distinct, stepwise increase in voltage was observed across the four states, providing a clear basis for discrimination.

The mean ± SD voltages were lowest for the gaseous state (257.12 ± 10.51 mV, 95% CI: 255.03–259.21 mV), followed by the solid state (398.12 ± 32.33 mV, 95% CI: 391.71–404.53 mV), the fluid state (507.24 ± 25.01 mV, 95% CI: 502.28–512.20 mV), and highest for the empty state (658.56 ± 14.85 mV, 95% CI: 655.61–661.51 mV) (Fig 7, Table 2).

**Table 2:** Voltage values of different colonic content states (n = 100).

State	Voltage Value ($\overset{―}{X}$± *S*，mV)	95%*CI*		Minimum Value	Maximum Value	Range
		Lower Bound	Upper Bound			
Gaseous	257.12 ± 10.51	255.03	259.21	238	275	37
Solid	398.12 ± 32.33	391.71	404.53	315	442	127
Fluid	507.24 ± 25.01	502.28	512.20	472	560	88
Empty	658.56 ± 14.85	655.61	661.51	636	688	52

F(3, 396) = 5766.29, *p* < 0.001. All post-hoc pairwise comparisons between groups were significant (LSD test, p < 0.001).

**Fig 7:**
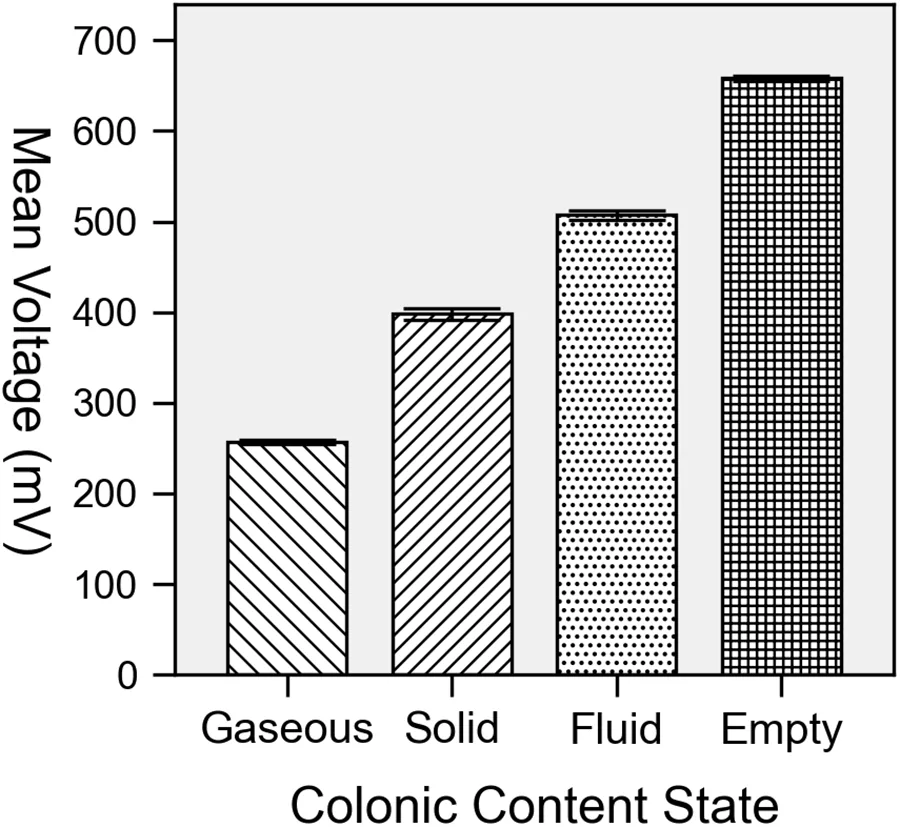
Voltage signatures of different in-vitro colonic content states.

Statistical analysis confirmed these observations. One-way ANOVA revealed a highly significant difference among the four groups (F (3, 396) = 5766.29, *p* < 0.001). Furthermore, post-hoc LSD comparisons demonstrated that all pairwise differences between the content states were statistically significant (*p* < 0.001 for all comparisons).

The bar chart displays the mean voltage signals recorded from the sensor in an in-vitro model under four different colonic content states: gaseous, solid, fluid, and empty. Error bars represent the 95% confidence interval (CI). Measurements were performed 100 times for each state (n = 100). Statistical analysis confirmed that the differences between all groups were significant (*p* < 0.001).

### 3. In-vivo validation of the smart sensing system

**(1) *O. cuniculus* model:** the system achieved a high overall classification accuracy of 98.0% across 400 total measurements (392 correct classifications). State-specific accuracies were excellent: 100% for the gaseous state, 99.0% for the empty state, 98.0% for the fluid state, and 95.0% for the solid state (Table 3). The system also provided clear visual feedback corresponding to each detected state (Fig 8). The 8 misclassification events were infrequent, with the majority (5 out of 8) involving the solid and fluid states being incorrectly identified as gaseous.

**Table 3:** In-vivo discrimination accuracy of the smart sensing system in the *O.cuniculus* model.

State	Misclassification				Correct Count
	Gaseous	Solid	Fluid	Empty	
Gaseous	0	0	0	0	100
Solid	3	0	2	0	95
Fluid	2	0	0	0	98
Empty	1	0	0	0	99

**Fig 8:**
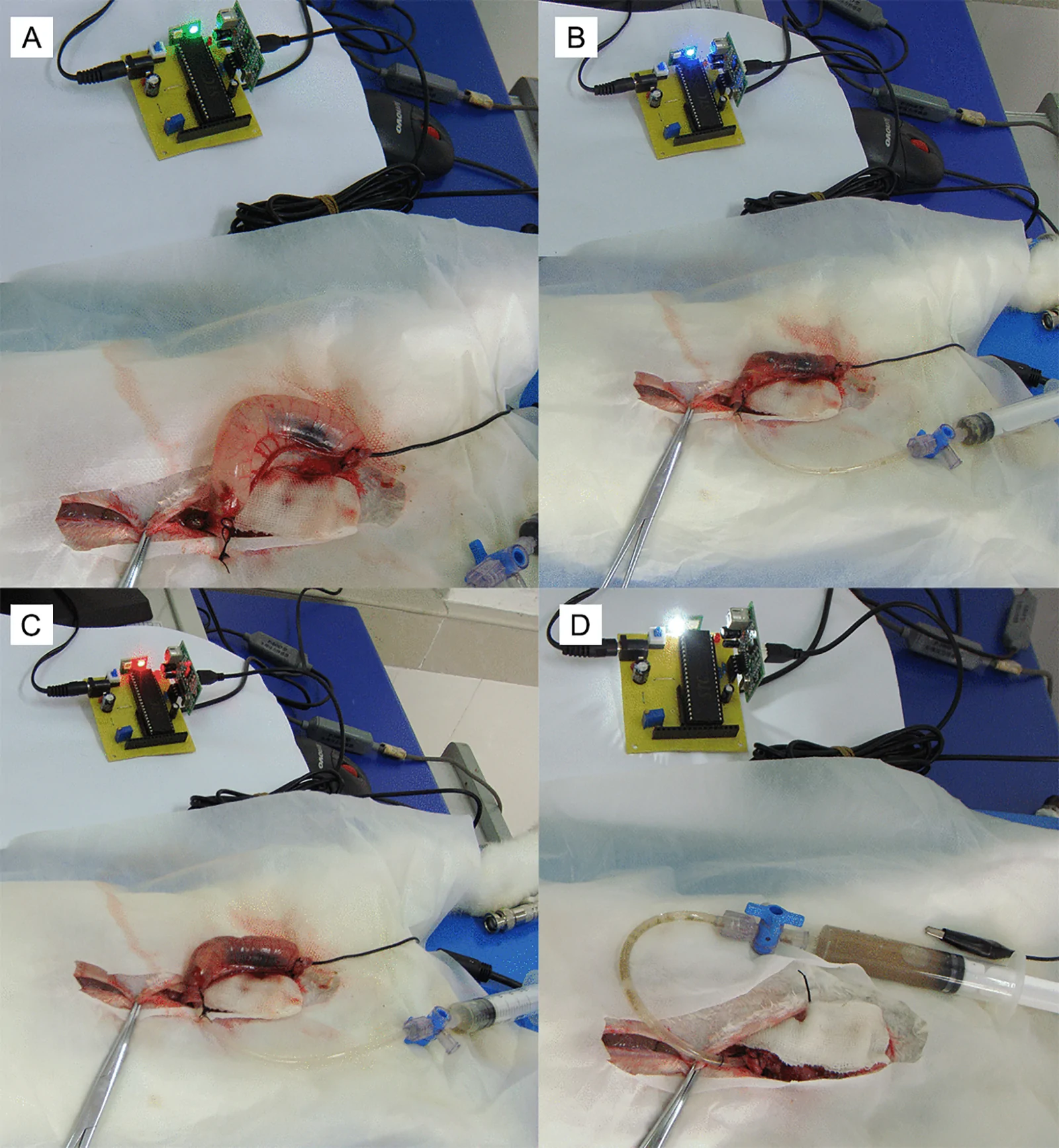
In-vivo visual feedback of the smart sensing system in the *O.cuniculus* model. Representative images from the in-vivo rabbit experiments demonstrate the system’s LED feedback for each detected colonic content state. The four-panel figure shows the system indicating the: **(A)** gaseous state with a green light, **(B)** solid state with a blue light, **(C)** fluid state with a red light, and **(D)** empty state with a white light.

**(2) *C. lupus familiaris* model:** The device maintained a high level of performance, achieving an overall accuracy of 97.0% (419 correct out of 432 classifications) across 432 total measurements. The state-specific accuracies remained high: 99.1% for gaseous, 97.2% for empty, 96.3% for fluid, and 95.4% for the solid state (Table 4). Consistent with the rabbit model, the solid state exhibited the lowest accuracy.

**Table 4:** In-vivo Discrimination Accuracy of the Smart Sensing System in the *C. lupus familiari*s Model.

State	Misclassification				Correct Count
	Gaseous	Solid	Fluid	Empty	
Gaseous	0	1	0	0	107
Solid	2	0	3	0	103
Fluid	3	0	0	1	104
Empty	2	0	1	0	105

## Discussion

Defecatory sensation plays a crucial role in anal continence, and the loss of this sensory function is a major contributing factor to fecal incontinence, which severely impairs the quality of life of ostomy patients [21]. The prerequisite for voluntary anal continence lies in the perception of defecatory urge, followed by subjective control of defecation to initiate bowel movements at an appropriate time [22]. Therefore, an ideal artificial anal plug should not merely be a passive barrier, but an active sensory device. In this study, the successful reconstruction of such sensory function offers a potential pathway toward continence management solutions that are more intelligent and responsive to patient needs.

Based on the physical properties of ultrasonic wave propagation, this study integrated modern microelectronic communication technology and intelligent feedback modules to successfully constructed a smart sensing system that exhibits a significant medium-dependent voltage stratification. The observed voltage elevation with increased immersion and across different colonic content states (from gaseous to empty) stems from the distinct reflection and attenuation behaviors of ultrasonic waves in media with varying acoustic impedances [19]. Specifically, lower acoustic impedance induces severe signal attenuation and weak reflected signals [23], corresponding to the minimal voltage in the non-immerse state, while higher acoustic impedance minimizes attenuation and eliminates scattering and diffraction interference [23], resulting in the peak voltage in the fully immersed states. Under the gas liquid interface, a significant impedance discrepancy generates intensified reflection [24] that manifests as intermediate voltage in the semi-immersed state. The returning ultrasonic signals are converted into electrical outputs and processed by the system circuitry, including rectification and filtering, which suppresses power-supply interference and ambient noise and improves signal stability. Collectively, acoustic impedance differences among gaseous, solid, fluid, and empty states generate distinguishable signals that can be translated into distinct characteristic voltage outputs, supporting reliable discrimination of colonic content states in our experimental setting.

In both in-vitro and in-vivo experiments, the sensing system exhibited robust discriminative performance. In vitro, voltage signals showed clear separation among the four colonic content states, which supported threshold setting for state discrimination. Using these thresholds, in-vivo validation in the *O. cuniculus* and *C. lupus familiaris* models demonstrated high classification accuracy based on LED and alarm outputs. The device consistently identified the absence of intestinal contents, achieving an accuracy of 99.0% (99/100) in the *O. cuniculus* model and 97.2% (105/108) in the *C. lupus familiaris* model for the empty state. This performance is practically important, because occasional false alarms are generally tolerable, whereas missed detections of intestinal contents may lead to leakage and should be avoided. Furthermore, it is noteworthy that even when misclassifications occurred between different content types (e.g., solid vs. fluid), the alarm was still triggered, indicating content presence and prompting user attention.

Regarding discrimination among different non-empty content states, the system showed the best performance for the gaseous state in vivo. In the *O. cuniculus* model, gaseous content was classified with 100% accuracy (100/100), and in the *C. lupus familiaris* model only one misclassification was observed (107/108; 99.1%). This outcome may be attributed to the effect of gas induced colorectal dilation, that reduces environmental interference [25]. By contrast, the solid and fluid states showed relatively lower accuracies in vivo (*O. cuniculus*: 95/100 and 98/100; *C. lupus familiaris*: 103/108 and 104/108, respectively). Misclassifications were mainly observed between solid and fluid, and in several instances toward the gaseous category. Potential causes of these misclassifications include residual air between the probe surface and fecal material, gas bubbles within feces or liquid stool, heterogeneous mixing of contents, and interference from intestinal fluid, all of which can alter local acoustic interfaces and consequently shift the reflected signal characteristics [26,27]. These findings suggest that distinguishing solid from fluid content is particularly challenging in vivo and may be improved by testing the system with a wider range of stool consistencies.

In the clinical management of fecal incontinence, traditional anal plugs have been proven to be an effective short-term intervention for symptom control, and multiple clinical studies have demonstrated that these plugs can reduce fecal leakage to a certain extent by leveraging a physical obstruction mechanism and improving patients’ quality of life. However, such plugs exhibit significant functional limitations: firstly, they lack the capability to sense the intestinal internal environment and cannot predict the presence or state of intestinal contents, forcing patients to rely on subjective sensations or scheduled removal for inspection, which not only compromises usability but also increases the risk of fecal leakage [7,28,29]. Furthermore, due to concerns about leakage, patients tend to repeatedly remove the plugs to check their internal conditions, significantly elevating the risk of peristomal skin complications [30]. There were relevant studies indicating that the overall incidence of peristomal skin complications can reach 43% [31], and some patients even have to discontinue use due to skin and subcutaneous tissue reactions [32]. A 2024 systematic review clearly stated that ostomy continence is closely related to patient care and quality of life [33], yet current traditional anal plugs still lack intelligent sensing modules and thus fail to achieve the precise regulation, resulting in the absence of a durable and reliable solution to date.

Beyond conventional anal plugs, several sensing approaches have been explored for bowel status monitoring, including pressure-based sensing [34,35], electrical impedance based sensing [36], and chemical or electrochemical sensing [37,38]. However, pressure signals can be confounded by peristalsis and body motion [39], whereas impedance and chemical sensors often face challenges related to contact dependence, biofouling, and signal drift in the colonic environment [40]. In this context, ultrasound represents an attractive alternative because it can differentiate content states based on acoustic contrast [41] while remaining feasible for miniaturized implementation with conventional signal processing [42],making it well suited for integration into an anal plug device. Accordingly, the ultrasonic sensing system developed in this study enables real-time identification of intestinal content presence and state, providing objective and actionable intraluminal feedback under physiologic conditions and potentially reducing reliance on subjective sensation or repeated plug removal for checking, which may help mitigate leakage risk and peristomal skin complications. This may be particularly relevant for individuals with reduced or unreliable defecatory sensation who rely on plug-based continence management, especially when stool consistency is unpredictable and timely awareness of content state is needed.

In support of this potential applicability, we validated the system in vivo in two animal models, indicating that the system can work not only under controlled conditions but also in a more realistic physiological setting. However, these results may not directly translate to humans, because bowel size, wall thickness, stool composition, and motility differ across species and can affect ultrasonic coupling and voltage readings. Therefore, the voltage thresholds established in animals should be viewed as animal-specific and would need to be re-established and validated before clinical use in humans.

From a translational perspective, the main implication of this study is that the voltage patterns identified in vitro can be implemented in a working device and still provide interpretable, real-time feedback in vivo, supporting the feasibility of further translational development. Moving from an experimental prototype to clinical use will require demonstrating that the sensor output and thresholds remain stable during long-term use, and that the device can operate safely in the colonic environment without loss of performance. In parallel, the hardware needs to be further miniaturized and reliably sealed with medically suitable materials, and the current external connections should be replaced by a practical power and alert solution that patients can use in daily life. Ultimately, human studies will be required to evaluate safety and usability and to determine whether the system can reduce clinically meaningful problems such as leakage events and frequent plug removal.

This study has several limitations. First, the simulated intestinal content states were simplified to four typical categories and did not include clinically common mixed or intermediate conditions, like semi-solid feces or mucus-mixed feces, limiting assessment of performance across the full range of real-world scenarios. Second, the current classification is based on fixed voltage cutoffs; improving the algorithm may help reduce errors, especially when distinguishing solid from fluid contents under complex in-vivo conditions. Finally, while interspecies differences and the need for human recalibration have been discussed above, additional work is still required to evaluate long-term stability and durability of the device and its signal performance under chronic use conditions.

## Conclusion

This study developed an integrated ultrasonic sensing system for artificial anal plugs. Distinct voltage signatures were observed in vitro across four colonic content states, supporting threshold-based state discrimination. In vivo validation achieved overall accuracy of 98.0% (*O. cuniculus*) and 97.0% (C. *lupus familiaris*), with 98.1% pooled accuracy for identifying the empty state. These findings suggest that the device can deliver real-time, state-specific feedback on intestinal contents, which may help inform timely user action.
